# *Leishmania donovani* Infection Enhances Lateral Mobility of Macrophage Membrane Protein Which Is Reversed by Liposomal Cholesterol

**DOI:** 10.1371/journal.pntd.0003367

**Published:** 2014-12-04

**Authors:** Moumita Ghosh, Koushik Roy, Dipanwita Das Mukherjee, Gopal Chakrabarti, Kingshuk Roy Choudhury, Syamal Roy

**Affiliations:** 1 Infectious Diseases and Immunology Division, CSIR-Indian Institute of Chemical Biology, Kolkata, India; 2 Department of Biotechnology, University of Calcutta, Kolkata, India; 3 Departmentof Biostatistics and Bioinformatics, Duke University, Durham, North Carolina, United States of America; McGill University, Canada

## Abstract

**Background:**

The protozoan parasite *Leishmania donovani* (LD) reduces cellular cholesterol of the host possibly for its own benefit. Cholesterol is mostly present in the specialized compartment of the plasma membrane. The relation between mobility of membrane proteins and cholesterol depletion from membrane continues to be an important issue. The notion that leishmania infection alters the mobility of membrane proteins stems from our previous study where we showed that the distance between subunits of IFNγ receptor (R1 and R2) on the cell surface of LD infected cell is increased, but is restored to normal by liposomal cholesterol treatment.

**Methodology/Principal Findings:**

We determined the lateral mobility of a membrane protein in normal, LD infected and liposome treated LD infected cells using GFP-tagged PLCδ1 as a probe. The mobility of PLCδ1 was computationally analyzed from the time lapse experiment using boundary distance plot and radial profile movement. Our results showed that the lateral mobility of the membrane protein, which is increased in infection, is restored to normal upon liposomal cholesterol treatment. The results of FRAP experiment lent further credence to the above notion. The membrane proteins are intimately linked with cellular actin and alteration of cellular actin may influence lateral mobility. We found that F-actin is decreased in infection but is restored to normal upon liposomal cholesterol treatment as evident from phalloidin staining and also from biochemical analysis by immunoblotting.

**Conclusions/Significances:**

To our knowledge this is the first direct demonstration that LD parasites during their intracellular life cycle increases lateral mobility of membrane proteins and decreases F-actin level in infected macrophages. Such defects may contribute to ineffective intracellular signaling and other cellular functions.

## Author Summary

The protozoan parasites, *Leishmania donovani*, replicate within the macrophages of the mammalian hosts. During its intracellular lifecycle, the parasite induces a wide variety of defects in the membrane homeostasis. Membrane bound receptor molecules are important for interacting with external stimuli. Our study very clearly showed that there is an increase in the mobility of membrane protein coupled with decrease in F-actin in infected cells, which may be corrected by liposomal cholesterol treatment. This observation indicates that intracellular parasite may alter the membrane biology of infected cells which may dampen overall cellular function.

## Introduction

The protozoa parasite, *Leishmania donovani*, (LD) replicates within the macrophage of the mammalian host [1]. The parasite during their intracellular life cycle causes wide variety of defects in cellular physiology like decrease in membrane cholesterol [2] with concomitant increase in membrane fluidity [3]. LD infectedmacrophage are unable to stimulate antigen specific T cells [3] and display defective IFNγ receptor 1 (R1) and receptor 2 (R2) subunit assembly [4]. Kala-azar patient showed progressive decrease in serum cholesterol as a function of splenic parasite load [5]. Interestingly, defective T cell stimulating ability and IFNγR assembly of infected macrophage can be corrected by liposomal cholesterol treatment [2], [4]. Cholesterol is one of the main constituent of the cell membrane and is important for raft assembly [6]. There are controversial reports on cholesterol depletion and membrane protein mobility, in some cases cholesterol depletion suppresses membrane protein mobility [7]–[9] while in others it increases the lateral mobility of the membrane protein [10], [11].

Several studies showed that decreased protein mobility on cholesterol depletion is due to the changes in the architecture of the underlying cytoskeleton [12], [13]. The formation of an immunological synapse between T cells and antigen presenting cells (APC) is recognized as a key event for activation of T cell [14]andactin cytoskeleton plays an important role in T cell activation [15]. Cholesterol, an important constituent oflipid raft [6], is intimately involved in the dynamics of immune synapse formation [16]. Previously we showed that splenic macrophages of infected hamster are incapable to form immunological synapse while splenic macrophages of liposomal cholesterol treated infected hamsters can form immunological synapse [2]. The duration of contact between T cell and APC is also important for T cell activation. The mature synapse lasts for several hours and is thought to be important for sustained signalling [17]. There is a report that slow moving peptide-MHC complex but not fast moving ones can form immunological synapse [18]. It has been shown in *Plasmodium falciparum* infection that the lateral mobility of erythrocytes membrane protein is related to the infective stages of the parasite [19].

1-phosphatidylinositol 4,5-bisphosphate phosphodiestares delta 1 (PLCδ1) is a membrane protein that catalyzes hydrolysis of phosphatidylinositol 4,5 biphosphate to generate diacylglycerol and inositol 1,4,5 triphosphate (IP3). Using PLCδ1 as a representative of membrane proteins,we show that under parasitized condition the lateral mobility of PLCδ1 is significantly increased coupled with reduced actin polymerization. The increased lateral mobility of PLCδ1 observed in LD infected macrophages is restored to normal upon liposomal cholesterol treatment. The enhance dynamic of membrane proteins may contribute to the defective signal transduction leading to defective cellular function.

## Materials and Methods

### Ethics statement

Use of mice was approved by the Institutional Animal Ethics Committee of Indian Institute of Chemical Biology, India. All animal experimentations were performed according to the National Regulatory Guidelines issued by CPSEA (Committee for the Purpose of Supervision of Experiments on Animals), Ministry of Environment and Forest, Govt. of India. The protocol number is SDR/SYR/2007.

### Reagents

LipoFECTAMINE, alexa 488 phalloidin and FCS (Fetal calf serum) were purchased from Invitrogen. Luria-Bertani media purchased from Merck, India. DNA preparation minikit was purchased from Qiagen. Penicillin-streptomycin, kanamycin, chloramphenicol, sodium bicarbonate, β mercapto-ethanol, RPMI-1640, M199, Hoechst 33258were purchased from Sigma Aldrich (St. Louis, MO). Cholesterol, phosphatidyl choline and cholesterol analogue were purchased from Avanti Polar Lipids. β-Actin antibody (mouse monoclonal IgG_1_) and secondary antibody (goat anti-mouse-HRP) were obtained from Santa Cruz and Bangalore Genei, Bangalore, India respectively. *plcδ1-gfp* plasmid is a kind gift of Dr. TamasBalla, NIH, USA.

### Cell line

RAW 264.7 (murine macrophage cell line) was used for *in vitro* experiments. For convenience RAW 264.7 was defined as macrophages (MΦ). The cell line was maintained in RPMI-1640 medium supplemented with 10% FCS and β-mercaptoethanol (5×10^−5^ M) at 37°C with 5% CO_2_ in a humidified atmosphere.

### Maintenance of *Leishmania donovani* (LD)

*Leishmania donovani *strain AG83 (MHOM/IN/1983/AG83), originally obtained from Indian kala-azar patients, was maintained in Golden Hamsters as described previously [20]. Promastigotes obtained after transforming amastigotes from spleen of infected animals were maintained in culture M199 supplemented with 10% FCS at 22°C. The culture was replenished with fresh medium every 72–96 h.

### Transfection of RAW264.7 cell line

RAW 264.7cells (10^4^ cells/well) were platedinto 8 chambered coverslips (BD Bioscience) for 14 h. The non-adherent cells were removed by washing. The cells were transfected with *plcδ1-gfp* plasmid using LipoFECTAMINE following the Invitrogen LipoFECTAMINE kit protocol. After 6 h, the cells were washed and complete medium was added.

### Infection of RAW 264.7 cell with LD

RAW 264.7 cells were infected as described previously [4]. Briefly RAW 264.7 cells (10^5^/10^6^) were allowed to adhere coverslips/petri dish for 24 h at 37°C under 5% CO_2_ atmosphere, after which the non-adherent cells were removed by gentle washing with serum-free medium. The adherent cells, after overnight incubation in complete medium, were challenged with stationary phase LD promastigotes at a cell to parasite ratio of 1∶10 and incubated for 6 h at 37°C. Excess parasites were then washed off with serum-free medium. The cells were then incubated further for 48 h. At end points the cover slips were washed with PBS, dried, fixed with 100% methanol, and stained with 10% Giemsa. The intracellular parasites were enumerated microscopically and the results were expressed as % infected RAW 264.7 cells as well as the number of parasites/100 RAW 264.7 cells. For convenience, LD infected RAW264.7 cells were defined as I-MΦ.

### Liposome preparation and treatment of LD infected RAW 264.7 cells

Liposomal cholesterol and liposomal cholesterol analogue were prepared using cholesterol/cholesterol analogue and phosphatidylcholine (PC) at a molar ratio of 1.5∶1 as previously described [2]. Briefly, 5.8 mg cholesterol/cholesterol analogue (4-cholestene-3-one) and 8 mg PC in chloroform were mixed and a thin film was prepared; subsequently, the film was dissolved in 1 ml saline and sonicated (Microson Ultrasonic cell disruptor with a Misonix 2-mm probe) at 4°C three times for 1 min each time at maximum output. The LD infected RAW264.7 cells (10^4^ cell/200 µl) were incubated with 10 µl liposomes for 20 h at 37°C. The cells were then washed three times in serum-free RPMI 1640 medium and finally resuspended in 10% FCS containing RPMI 1640. For convenience, liposomal cholesterol treated LD infected cells as I-MΦ-CL, and liposomal cholesterol analogue treated LD infected cells as I-MΦ-AL.

### Confocal live cell imaging

The *plcδ1-gfp* transfected RAW 264.7 cells were visualized under Confocal microscope. The cells were incubated with 1 µg/ml Hoechst 33258 solution for 2 minutes and washed off with PBS (Phosphate buffered saline, pH 7.2) to visualise the nucleus. The cells were then kept in complete medium (RPMI 1640 containing 10% FCS) and live cell images of the cells were taken for 1 minute with 8 seconds interval. GFP was imaged using a wide field microscope (model: Andor Spinning Disc Confocal Microscope, Olympus). The fluorochrome was excited with a mercury arc lamp. For alexa 488 excitation and emission wavelength was488 and 525 nm. For Hoechst 33258 these were 405 and 447 nm respectively. Images, obtained using identical exposure times for cells subjected to various treatments in each experiment, were collected using a 60X1.42 NA Plan-ApoN objective and captured using Andor IXON897EM CCD camera.

### Computational analysis of spatial distribution of PLCδ1-GFP

The spatial distribution of the expression of PLCδ1-GFP in normal, infected and liposomal cholesterol treated infected cells was computationally analyzed using boundary distance analysis as described previously [21]. Briefly, the differences in the shape and size of the cells were adjusted by normalizing the spatial co-ordinates of the cell. The normalization were done on a one dimensional scale with the following features: (i) the ‘center’ of the cell has a radial distance of 0 (ii) points on the boundary of a cell to be a radial distance of 1 (iii) points in the interior of a cell have a radial distance between 0 and 1, depending on how close they are to the boundary of the cell (iv) points outside the boundary of the cell have a radial distance of more than 1, with further points having greater distance. The co-ordinate system was computed separately for each cell.

### Measurement of mobility of PLCδ1-GFP

Detecting the motion of the PLCδ1-GFP at a given location in a cell is a hard problem because we cannot track individual particles over time. From live cell imaging, we obtained a time sequence of images, in a short space of time (1 minute), each of which the expression distribution has changed slightly from the previous one. From this sequence, we measured the temporal flux, in GFP expression, as follows
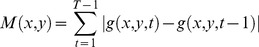
(1.1)Where *g*(*x,y,t*) is the expression of the PLCδ1-GFP at location (*x,y*) and timepoint *t*. The movement (flux) measured, *M*(*x,y*) depends on how much the expression level changes from one timepoint to the next. In order to resolve the spatial distribution of movement, we have used the radial mapping concept described in the previous section for (1.1), but this time mapping the movement measure *M*(*x,y*) instead of the expression levels. For increased comparability, we again normalized the movement measure by the overall level of expression for each cell.

### Measurement of PLCδ1-GFP peak level expression

The highest level of expression or peak expression of GFP occurs at the membrane for each cell. Thus we calculated P, peak level of expression of GFP for each cell. To compare these measurements across groups, we fitted a model of the form
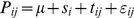
(1.2)Where *P_ij_* is the peak expressions of GFP for cell *j* in study *i*. The model terms include *μ*, the baseline mean for N-MΦ and *s_i_*, an effect due to imaging study *i*. Since the factors that can influence the imaging study, such as ambient lighting, level of *plcδ1-gfp* transfection etc., are unmeasured, we assume that this effect is random and has a zero mean Gaussian distribution. The main quantity of interest is *t_ij_*, the effect of infection or treatment and finally *ε_ij_* is measurement error, assumed to have an independent mean zero Gaussian distribution. The Parameter estimates and hypothesis tests were carried out by fitting the corresponding mixed effects model using the R computing platform (www.r-project.org).

### Fluorescence Recovery after Photobleahing (FRAP)

FRAP measurement was done as described [22]. Briefly, GFP was imaged using a wide field microscope (model Andor Spinning Disc Confocal Microscope, Olympus). The fluorochrome was excited with a mercury arc lamp, using excitation wavelength of 488 nm; the emission wavelength was 525 nm. Cells were kept at 37°C in a humidified chamber. A 60X 1.42 NA Plan-ApoN objective was used with the confocal pinhole set at 1–2 Airy units. Photobleaching of GFP was performed with the 488-nm laser line at 30 mW power in a 32.85 µm^2^ rectangular region of interest. Pre- and post-bleach images were monitored at low laser intensity (14 mW). Fluorescence recoveries in the bleached region and overall photobleaching in the whole cell during the time series were quantified using the iQ2.7 software. In the confocal microscope, a prebleach series (usually about 10 images) at low illumination was acquired to measure the fluorescence equilibrium before photobleaching. One or more spots or regions of interest (ROI) were illuminated with high intensity to photobleach the area of interest. Finally, a post bleach image series were acquired to measure the recovery kinetics of fluorescent light intensity. The images were captured continuously (real time) for 30 s. Mobility of the PLCδ1-GFP were extracted from equation (1.3).
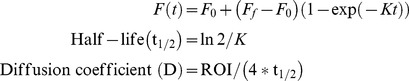
(1.3)where ROI is the Region of Interest, *F*(*t*) is the intensity of ROI at time *t*, *F*_0_ is the intensity of ROI immediately after bleach, *F_f_* is the intensity of ROI at saturation, *K* is the recovery rate constant.

### Confocal imaging of actin cytoskeleton

The cells were permeabilized and fixed with 2% paraformaldehyde containing 0.1% Triton X 100. To visualize actin, the cells were then stained with alexa 488 conjugated phalloidin for 30 min as described by manufacturer. After, washing the cells were mounted on a mounting medium containing DAPI. Alexa 488 fluorescence was imaged using a wide field microscope (model Andor Spinning Disc Confocal Microscope, Olympus). The fluorochrome was excited with a mercury arc lamp. For alexa 488 excitation and emission wavelength was 488 nm and 525 nm respectively. Similarly for DAPI, excitation and emission wavelengths were 405 nm and 447 nm respectively. Images were collected using a 60X 1.42 NA Plan-ApoN objective and captured using Andor IXON897EM CCD camera. These were obtained using identical exposure times for cells subjected to various treatments in each experiment.

### F-actin fractionation and western blot analysis

The cellular F-actin (polymerized form) was isolated by modifying the method described previously [23]. Briefly, the cells were pelleted by centrifugation at 14000 rpm and resuspended in actin stabilisation-extraction buffer (0.1 M Pipes pH 6.9, 30% glycerol, 5% DMSO, 1 mM MgSO_4_, 10 µg/ml antiprotease cocktail, 1 mM EGTA, and 1% Triton X-100) at room temperature for 20 min and centrifuged at 14000 rpm for 15 min [24]. The pellet comprising the F-actin was dissolved in actin extraction buffer (2 mMTris-HCl pH-8, 1 mM Na_2_.ATP, 0.2 mM CaCl_2_, 0.5 mM DTT) [25]. For assaying total cellular actin, the cells were lysed with 1X RIPA Buffer from Cell signalling technology, Danvers, MA (20 mMTris-HCl pH 7.5, 150 mMNaCl, 1 mM Na_2_EDTA, 1 mM EGTA, 1% NP-40, 1% sodium deoxycholate, 2.5 mM sodium pyrophosphate, 1 mM beta glycerophosphate, 1 mM Na_3_VO_4_, and 1 mg/ml leupeptin, with recommended addition of 1 mM PMSF immediately before use) and centrifuged at 14000 rpm for 15 min [4]. The protein concentration was estimated by Bradford method using a Protein Estimation Kit (Merck Genei, Mumbai, India). For immunoblotting, equal amounts of protein were loaded to SDS-PAGE (10% gel) and electrotransferred to nitrocellulose membranes (Millipore, Bangalore, India) in a transfer buffer consisting of 20 mMTris-HCl, 150 mM glycine, and 20% methanol. Membranes were blocked overnight at 4°C in 5% BSA, probed with primary antibody against β-actin (1∶1000 dilution, mouse monoclonal IgG_1_, Santa Cruz) for 4 h at room temperature, and incubated with HRP-conjugated secondary antibody (1∶1500 dilution, Genei, Bangalore, India) for 2 h at room temperature. The chemiluminescence signal was detected using Super Signal West Pico Chemiluminescent Substrate (Pierce, Rockland, IL). The F-actin and total actin expressions were analyzed by image-J.

## Results

### Infection of RAW264.7 cells with *Leishmania donovani* (LD)

**Figure 1:**
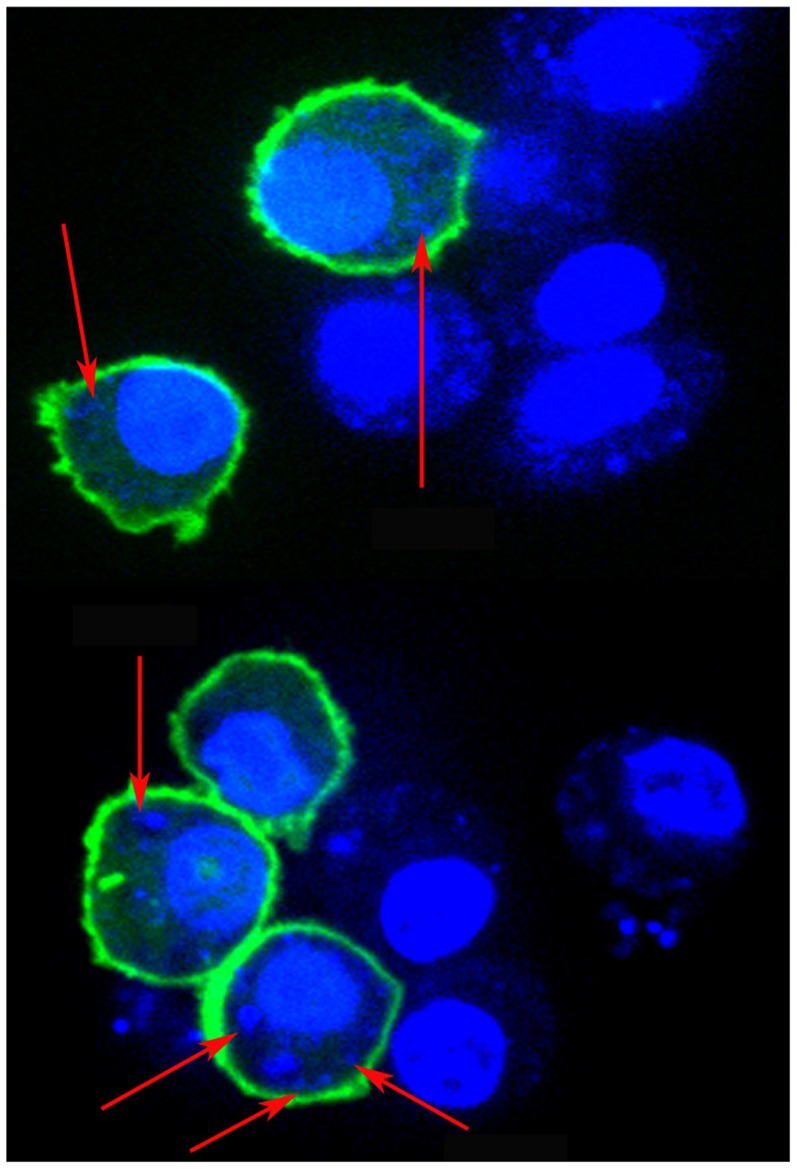
Confocal images of *plcδ1-gfp* transfected RAW 264.7 cells followed by infection with LD (I-MΦ). The expression of PLCδ1-GFP (green) is on the cell surface. The cells were stained with Hoechst 33342 (blue). Parasites are marked by blue dot and indicated by arrow.

For this investigation RAW264.7 cells were used as host cell for infection with LD. The number of intracellular LD and integrity of the nucleus in infected MΦ (I-MΦ) were determined by staining with Hoechst 33342 or DAPI. The intracellular parasite number, verified by Giemsa staining (S1 Figure), was ∼8–9/cell, similar to the results obtained by Hoechst 33342 or DAPI stain (Fig. 1). It was clear that the morphology of the cells and the integrity of nucleus did not change upon infection.

### Transfection of macrophage (MΦs) with *plcδ1-gfp*

Normal MΦs (N-MΦ) were transfected with *plcδ1-gfp* plasmid and the distribution of GFP was measured by confocal microscope. It was observed that green fluorescence was localized predominantly on the cell surface indicating cell surface expression of PLCδ1 (S2A Figure). N-MΦs were transfected with *plcδ1-gfp* plasmid and then infected with LD. The expression of PLCδ1 in I-MΦwas found to be localized predominantly on the cell surface (Fig. 1). Similarly expression of PLCδ1 in liposomal cholesterol treated infected macrophages (I-MΦ-CL) was predominantly localized on the cell surface (S2B Figure).

### Analysis of PLCδ1 expression

**Figure 2:**
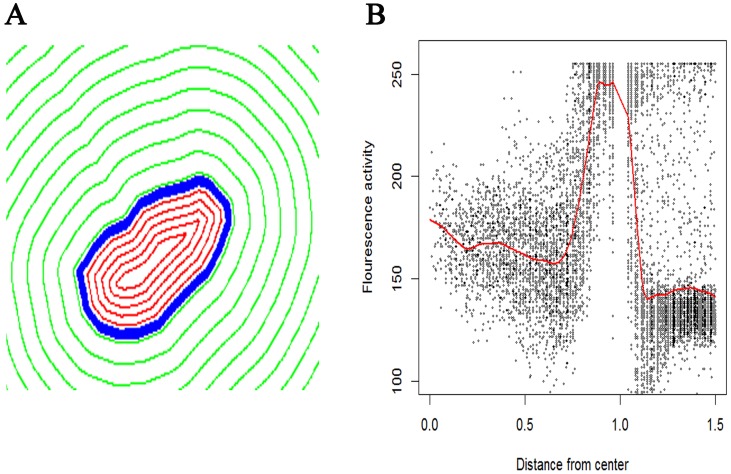
Radial distance map of a cell. A) Blue line shows cell boundary (radial distance = 1). Red line show contours of equal radial distance within the cell (radial distance <1). Green lines show contours of equal radial distance outside the cell (radial distance >1). B) Plot of the distribution of PLCδ1-GFP expression as a function of distance from center of the cell. The red line shows the average activity as a function of distance, obtained via a smoothing line. The x-axis (radial distance) is truncated at 1.5 to avoid contamination of PLCδ1-GFP expression from other cells.

**Figure 3:**
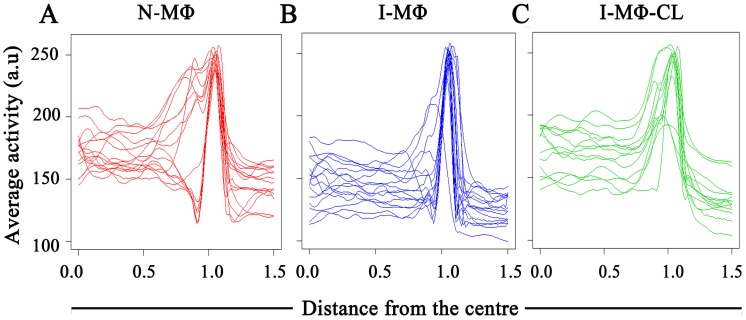
Analysis of PLCδ1 expression. RAW264.7 cells were transfected with *plcδ1-gfp* plasmid. Average expressions (activity) of the PLCδ1-GFP were measured by computational analysis as a function of distance from center of cell. Each line represents a particular cell. Activity is on a scale of 0–255 (arbitary unit, a.u.). A) N-MΦ; B) I-MΦ; and C) I-MΦ-CL.

The expression of PLCδ1 in N-MΦ, I-MΦ and I-MΦ-CL was quantified by computational method. The boundary distance co-ordinates were computed for each cell using the Euclidean distance transform after presmoothing and oversampling of cellular boundaries to normalize with respect to variations in cell shape and size. The general shape profile based on GFP expression in N-MΦ, I-MΦ and I-MΦ-CL were similar (Fig. 2A). An adaptive piecewise linear model is used to compare expression gradients in intra, peri and extra cellular zones.We have considered the center of the cell as ‘0’ and the cell surface as ‘1’ on the x-axis. The distribution of PLCδ1 are relatively flat in the interior of the cell (between 0 and 1 on the axis), followed by a sharp rise at the plasma membrane (at 1 on the x-axis) and finally a sharp drop off (points >1 on the x-axis) outside the cell (Fig. 2B). The average expression curves show the same basic spatial distribution in N-MΦ, I-MΦ and I-MΦ-CL: low near the center of the cell, peak near the boundary and then a drop to ∼‘0’ some distance outside the boundary (Fig. 3).

Differences in the highest level of PLCδ1expression or peak expression “P” across the cell was analyzed using the model in equation (1.2). There was some variation in the location of the PLCδ1 expression peak across cells: this could be due to shape deformable cell movement observed during live cell imaging. The expressions of PLCδ1 on the cell surface of N-MΦ, I-MΦ and I-MΦ-CL were 248.97, 241.76 and 236.04 a.u. respectively (Table 1). It appears that there was no significant difference in the expression of PLCδ1 in the above cell type.

**Table 1:** Analysis of PLCδ1-GFP expression at the boundary of cells for N-MΦ, I-MΦ and I-MΦ-CL was measured by computational analysis using equation (1.2).

	Expression (a.u.)	SE	DF	t-value	p-value
**N-MΦ**	248.97	7.62	28.00	32.67	0.00
**I-MΦ**	241.76	10.35	13.00	−0.70	0.50
**I-MΦ-CL**	236.04	10.78	13.00	−1.20	0.25

SE = standard error; DF = degree of freedom; t-value = t-test between the N-MΦ and other group; p-value = probability of obtaining test statistic.

### Analysis of PLCδ1 movement

**Figure 4:**
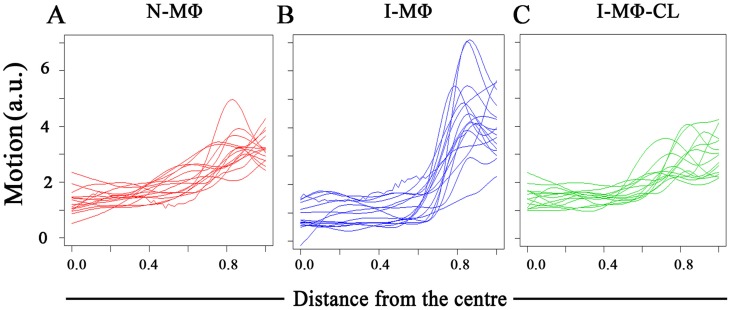
Analysis of PLCδ1 movement. RAW264.7 cells were transfected with *plcδ1-gfp* plasmid. Average motions of the PLCδ1-GFP were measured by computational analysis as a function of distance from center of cell. Each line represents a particular cell. Motion is on a scale of 0–6 (arbitary unit, a.u.). A) N-MΦ; B) I-MΦ; and C) I-MΦ-CL.

We studied the lateral mobility of PLCδ1 in LD infection using computational analysis. The radial profile of PLCδ1 movement showed that the movement is highest near the cell membrane irrespective of infection status of the cell. The highest movement at the cell boundary is denoted as peak movement or “P”. It was observed that peak movement is higher in the I-MΦ as compared to N-MΦ,whereas in I-MΦ-CL it was comparable to that in N-MΦ (Fig. 4). To confirm this result, we carried out an ANOVA analysis of PLCδ1 movement across cells, as described in equation (1.2). The fitted model indicates that movement of PLCδ1 at the cell membrane is significantly higher in I-MΦ as compared to N-MΦ (p-value = 0.04) and there was no significant difference between N-MΦ and I-MΦ-CL (p-value = 0.72) (Table 2).

**Table 2:** Analysis of the PLCδ1-GFP movement at the boundary of cells as measured by computational analysis using equation (1.1)for N-MΦ, I-MΦ and I-MΦ-CL.

	Flux (a.u.)	SE	DF	t-value	p-value
**N-MΦ**	4.43	0.29	28.00	15.32	0.00
**I-MΦ**	5.39	0.41	13.00	2.33	0.04
**I-MΦ-CL**	4.28	0.42	13.00	−0.37	0.72

The movement is indicated by flux value. Analysis was done by fitting model (1.2). SE = standard error; DF = degree of freedom; t-value = t-test between the N-MΦ and other group; p-value = probability of obtaining test statistic.

### Quantitative analysis of lateral mobility of PLCδ1 by FRAP

**Figure 5:**
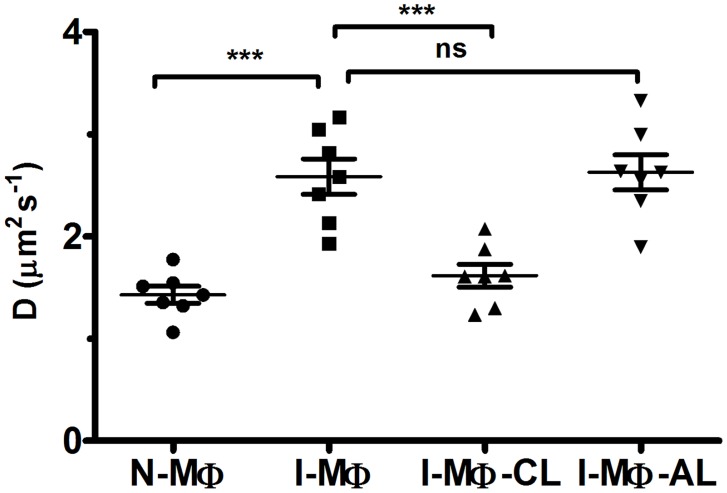
Lateral mobility of PLCδ1. Fluorescence recovery after photo bleaching was measured in *plcδ1-gfp* transfected RAW 264.7 cells. The diffusion Coefficient (D) of PLCδ1-GFP for N-MΦ, I-MΦ, I-MΦ-CL and I-MΦ-AL was measured using equation (1.3). The data represents average of 7 cells with ±SD. *** represents p<0.0005 and ‘ns’ means not significant.

The lateral mobility of PLCδ1 wasstudied in live cells at 37°C.The cells were bleached and fluorescence recovery of the bleached region was measured up to 30 s (S3A Figure). It was observed that the extent of recovery was similar in N-MΦ, I-MΦ, I-MΦ-CL and I-MΦ-AL (S4 Figure) though the rate of recovery was different. The analysis of fluorescence recovery kinetics showed that the diffusion coefficient of PLCδ1 in N-MΦ, I-MΦ, I-MΦ-CL and I-MΦ-AL was 1.4±0.2, 2.5±0.5, 1.6±0.3 and 2.6±0.5 µm^2^ s^−1^ respectively (Fig. 5). The photobleach effect was specific because in the unrelated region, mean fluorescence intensity was constant. To show that the recovery was predominantly from the plasma membrane, cytosolic protein was also bleached as a control. It was observed that the recovery of PLCδ1 was very poor almost not detectable (S3B Figure).

### Organizational analysis of actin filament

**Figure 6:**
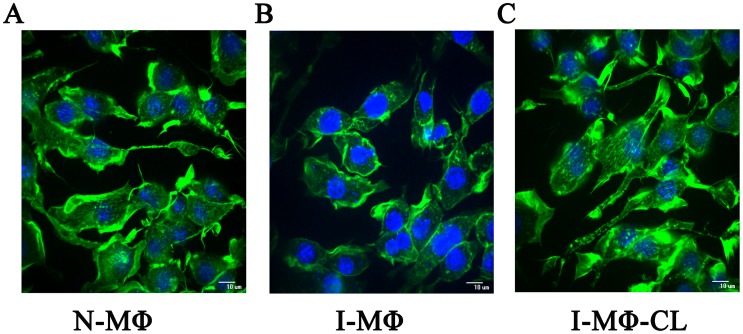
Alteration in actin cytoskeleton. The actin cytoskeleton change was determined by staining with alex488 conjugated phalloidin (green). Phalloidin binds to F-actin. The nucleus and parasite was stained with DAPI (blue) and visualized by confocal microscopy. A) N-MΦ, B) I-MΦ, and C) I-MΦ-CL.

We studied the actin cytoskeleton in N-MΦ, I-MΦ and I-MΦ-CL. The integrity of the cytoskeleton protein actin was measured by confocal microscopy after staining with fluorescence label phalloidin, which binds to filamentous actin (F-actin). The actin filamentare clearly visible in N-MΦ (Fig. 6A). However, in I-MΦactin filaments were reduced; insteadpossible actin depolymerisationwas noted (Fig. 6B). Interestingly, I-MΦ-CLdid display actin filaments (Fig. 6C). The computational analysis of phalloidin staining showed about 25% decrease in F-actin in I-MΦ and 9% increase in I-MΦ-CL as compared to N-MΦ. We next quantified F-actin by western blot and the result was expressed with respect to total actin. It was observed that the total actin level was similar in N-MΦ, I-MΦ and I-MΦ-CL (S5B Figure). Though F-actin level was reduced in I-MΦ, it reappeared upon liposomal cholesterol treatment (S5A Figure). There is about 30% decrease of F-actin in I-MΦ as compared to N-MΦ and in I-MΦ-CL F-actin was comparable (S5C Figure).

## Discussion

The relation between cholesterol depletion from the membrane and lateral mobility of the membrane proteins remains a contentious issue. There are reports to suggest that the mobility of membrane proteins is decreased upon cholesterol depletion [7]–[9], which may be due to formation of solid gel-like cluster in the membrane [26]. Again an increase in lateral mobility of membrane proteins like CD44 and wild type-H-Ras is reported after cholesterol depletion (10–12). There is an interesting observation in neuronal cells where lateral mobility of nicotinic acetylcholine receptor was found to be governed by the receptor composition and local domain and cell type [27].

Here we studied the lateral mobility of PLCδ1 as prototype of membrane protein. PLCδ1 is a raft associated protein having cytoskeleton interacting ability [28]. It is a well characterized protein in terms of domain sequences and membrane interacting domains. UNIprot entry of PLCδ1 from *Rattusnorvegicus* (ID: P10688) suggests existence of an N terminal pleckstrin homology (PH) domain (region 21–130) followed by two EF-hand domains (region 140–211) and two Phosphatidylinositol-specific phospholipase C domains (region 296–609) [29]. The C-terminal end of PLCδ1 is also flanked by a C2 domain (region 630–720) [30]. Both PH and C2 domains are known to involve in targeting proteins to cell membranes [31]. The transfection of *plcδ1-gfp* is well known to be specifically localized to plasma membrane with very little localization in the cytosol and nucleus [32].

The disease visceral leishmaniasis is characterized by immune suppression. Previously we showed that there is a defective synapse formation between T cells and parasitized MΦ [3]. The T-cell receptor recognizes Peptide-MHC complex in the context of antigen presenting cells (APC) like macrophages and dendritic cells [33]. The duration of contact between T-cells and APCs is critical for T-cell activation. It is an exciting proposition to study the lateral mobility of peptide-MHC complex to explain defective T-cell function in leishmaniasis. But there are issues that tend to discourage undertaking such studies. The infected macrophages show decreased affinity of MHC II towards peptide [34]; thus it will be difficult to interpret the defective T-cell stimulation due to increase in lateral mobility of MHC or lack of immunogenic peptides in association with MHC II protein. Furthermore there is a report on the existence of two species of peptide-MHC complex; the slower complex can form effective synapse but not the faster moving one [18]. It may be recalled that MHC class I when coupled with GFP (GFP-tagged H-2L^d^) shows relatively low diffusion coefficient [35]. The diffusion coefficient may be influenced by the protein dimensions [36]. In this context PLCδ1 offered advantages as it is a single chain membrane protein as opposed to MHC protein which contains two chains.

Our study showed that transfection of *plcδ1* led to over expression of PLCδ1 in the membrane of RAW 264.7 cells, similar to that reported in CHO cells by others [37]. In the latter case, despite substantial increase in PLCδ1 in membrane, IP3 production was only marginally increased and it still needed a stimulus to generate IP3 [37]; therefore it is unlikely that the lipid composition in transfected cells may be altered in our case.

Two complementary techniques have been exploited in this investigation to enhance our understanding in membrane protein dynamics under parasitized condition. It was observed that radial profile of PLCδ1 movement showed an increase in I-MΦ which was restored to normal upon liposomal cholesterol treatment (Fig. 4). This methodology has a limitation because we cannot track individual particles over time, as monitoring the motion of PLCδ1 at a given location in a cell is not easy [21]. FRAP study also showed an increase in the diffusion coefficient of PLCδ1 in I-MΦ as compared to N-MΦ (Fig. 5). The treatment of infected cells with liposomal cholesterol but not with liposomal cholesterol analogue (4-cholestene-3-one) restored lateral mobility of PLCδ1 (Fig. 5). The amphiphilic properties of cholesterol are provided by the hydrophilic 3β-hydroxy group and the hydrophobic tetracyclic ring with the isooctyl side chain at C-17. The reason we have included 4-cholestene-3-one as analogue of cholesterol is because it lacks the OH function of cholesterol which forms hydrogen bond with amide of sphingolipids, important for alignment of cholesterol in the membrane [38]. There was another study from our group where we showed that liposomal cholesterol but not the analogue of cholesterol favors raft assembly in infected macrophages [3]. The importance of 3β-hydroxyl function was further substantiated from another study by our group where we could show that the assembly of IFNγ receptor subunits (R1 and R2) on the surface of leishmania infected macrophages could only be restored by liposomal cholesterol, not by cholesterol analogue or DPPC-liposomes [4]. Unlike the exquisite specificity of 3β-hydroxyl function of cholesterol, side chain modification appears still permissible because cholesterol may be replaced by desmosterol for packing in the membrane [39].

Several studies showed the effect of cholesterol on mechanical properties of a cell through the underlying cytoskeleton [12], [13]. Changes in membrane cytoskeleton adhesion are expected to have a major impact on numerous cell functions [40]. The role of cytoskeleton meshwork as a barrier for lateral mobility of transmembrane proteins was established based on the findings that disorganized cytoskeleton meshwork favors faster lateral diffusion [41]. Kwik and coworkers [42] demonstrated that decrease in membrane protein mobility is associated with changes in architecture of the underlying actin network. F-actin plays an important role in restricting lateral mobility of membrane proteins in neuronal [43] and other cell types [44], which indicates direct or indirect tethering of membrane proteins to the cytosketeton. Quantification of such interaction using optical technique, i.e. TIFR may be of great use as reported by Huhn and Pollard, but such method is restricted to purified actin [45]. Here we show the effect of cytoskeleton actin filament disruption on LD infection and its restoration by liposomal cholesterol treatment (Fig. 6). We quantified F-actin by western blot and the result is expressed with respect to total actin. It was observed that total actin is essentially similar in N-MΦ, I-MΦ and I-MΦ-CL; F-actin is reduced (30%) only in I-MΦ but is restored upon liposomal cholesterol treatment (S5A–B Figure). The comptutational analysis of phalloidin staining showed that there is about 25% decrease in F-actin in I-MΦ and which is restored to normal in I-MΦ-CL. Therefore these two methods are in agreement.

The cause of actin depolymerisation in LD infection is unknown. It is tempting to speculate that intracellular LD by some unknown mechanism inhibits actin filament formation. Now the question comes how liposomal cholesterol treatment favors formation of actin filament. Recently we showed that liposomal cholesterol killed intracellular parasites [46]. Thus it may be possible that liposomal cholesterol treatment kills intracellular LD which may favor restoration of actin filament formation. The disruption of actin cytoskeleton may help to release the anchorage between cytoskeleton and membrane protein, making the movement of PLCδ1 free of cytoskeletal hindrance in LD infection. It may be recalled that a number of *Salmonella* strains carry *spv* virulence locus encoding the SpvB protein, an ADP-ribosyl transferase, which acts during intracellular infection to depolymerize the actin cytoskeleton [47]. There is also a report that *Toxoplasma gondii* infection changes the actin cytoskeleton of the dendritic cells due to secretion of parasite rhoptry [48].

In conclusion, this is the first report to our knowledge on changes in the cytoskeleton protein actin, coupled with increased lateral mobility of membrane protein in LD infected cells, which may have strong implication in altered plasma membrane architecture and defective signal transduction in infected macrophage.

## Supporting Information

Figure S1 Images of I-MΦ stained with Giemsa. The intracellular parasites were enumerated microscopically and the results were expressed as % infected RAW 264.7 cells as well as the number of parasites/100 RAW 264.7 cells.For convenience, LD infected RAW264.7 cells were defined as I-MΦ.The parasites are indicated by arrow. ∼70–80% cell were infected with ∼8–9 parasite per infected cells.
(TIF)

Figure S2 Confocal images of *plcδ1-gfp* transfected A) N-MΦ and B) I-MΦ-CL. The expression of PLCδ1-GFP (green) is on the cell surface. The cells were stained with Hoechst 33342 (blue).
(TIF)

Figure S3 FRAP of *plcδ1-gfp* transfected N-MΦ. A) The bleach of PLCδ1-GFP (green) on the cell surface. B) The bleach of PLCδ1-GFP (green) in the cytosol (control). Left panel, image of pre-bleach cell i.e. image of cell before bleach. Middle panel, image of cell at the time of bleach. Right panel, image of post-bleach cell i.e. image of cell 30 sec after bleach. Photobleaching of GFP was performed with the 488-nm laser line at 30 mWatpower in a rectangular region of interest 32.85 µm^2^. The bleach areas are indicated by red rectangle.
(TIF)

Figure S4 Fluorescence recovery of PLCδ1-GFP in N-MΦ (black circle), I-MΦ (red-square), I-MΦ-CL (green triangle) and I-MΦ-AL (blue triangle) after photo bleach. The data are expressed as % of recovery.
(TIF)

Figure S5 Immunoblot of actin. A) Expressions of F-actin. B) Expressions of total cellular actin. 10^6^/ml cell were lysed in a RIPA buffer. 100 µg of cellular protein were used to detect F-actin and total cellular actin. C. The densitometry ratio of F-actin/total actin. The analysis was done by image-J.
(TIF)
