# Correction: *Leishmania donovani* Infection Enhances Lateral Mobility of Macrophage Membrane Protein Which Is Reversed by Liposomal Cholesterol

**DOI:** 10.1371/journal.pntd.0004640

**Published:** 2016-04-13

**Authors:** Moumita Ghosh, Koushik Roy, Dipanwita Das Mukherjee, Gopal Chakrabarti, Kingshuk Roy Choudhury, Syamal Roy

There is an omission in the Funding Statement. The correct funding information is as follows: This work was supported by Council of Scientific and Industrial Research (New Delhi): Network Projects BSC 0114 and BSC 0120. MG, KR and DDM are thankful to CSIR for fellowship. The funders had no role in study design, data collection and analysis, decision to publish, or preparation of the manuscript.
