# Supplementary figures and images for: Leishmania donovani Infection Enhances Lateral Mobility of Macrophage Membrane Protein Which Is Reversed by Liposomal Cholesterol
Source: PLoS Negl Trop Dis. 2014 Dec 4;8(12):e3367. doi: 10.1371/journal.pntd.0003367 (PMC4256160; doi:10.1371/journal.pntd.0003367)

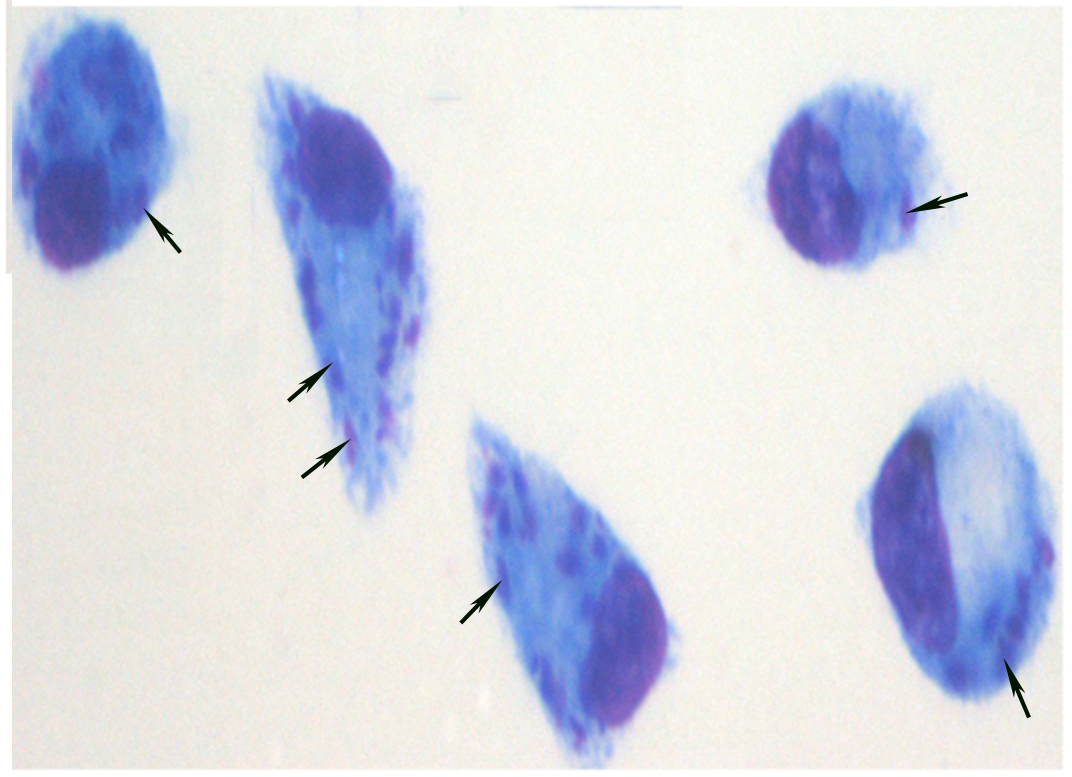

Supplement: Figure S1 — Images of I-MΦ stained with Giemsa. The intracellular parasites were enumerated microscopically and the results were expressed as % infected RAW 264.7 cells as well as the number of parasites/100 RAW 264.7 cells.For convenience, LD infected RAW264.7 cells were defined as I-MΦ.The parasites are indicated by arrow. ∼70–80% cell were infected with ∼8–9 parasite per infected cells. (TIF) [file pntd.0003367.s001.tif]

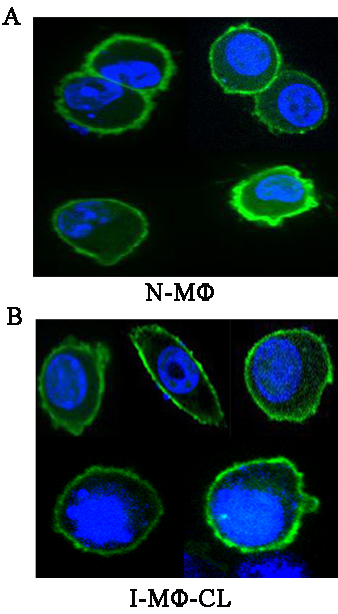

Supplement: Figure S2 — Confocal images of plcδ1-gfp transfected A) N-MΦ and B) I-MΦ-CL. The expression of PLCδ1-GFP (green) is on the cell surface. The cells were stained with Hoechst 33342 (blue). (TIF) [file pntd.0003367.s002.tif]

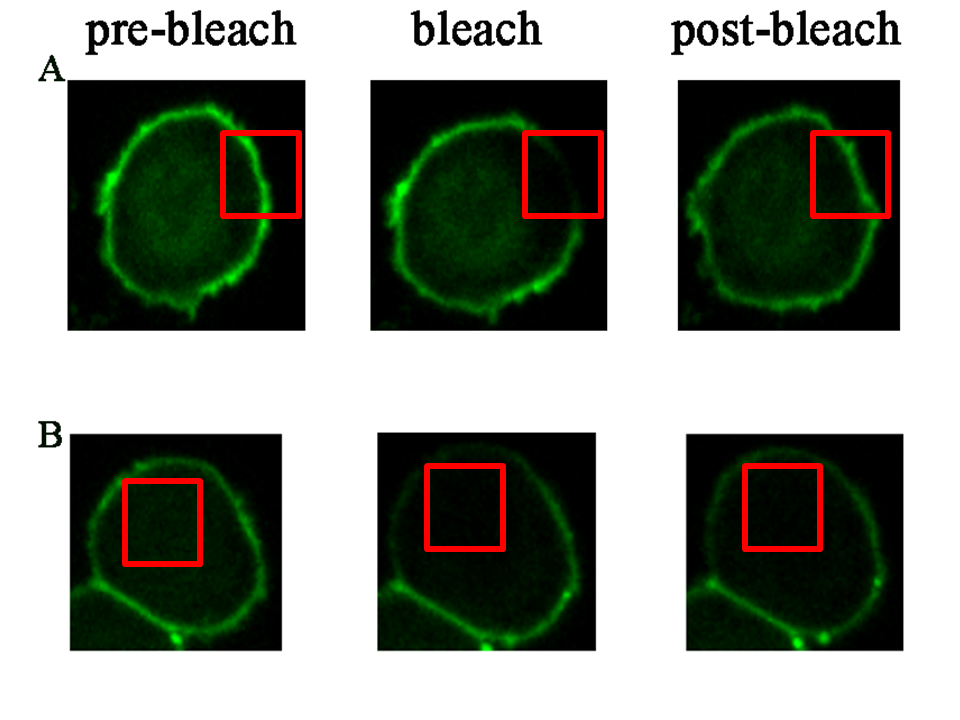

Supplement: Figure S3 — FRAP of plcδ1-gfp transfected N-MΦ. A) The bleach of PLCδ1-GFP (green) on the cell surface. B) The bleach of PLCδ1-GFP (green) in the cytosol (control). Left panel, image of pre-bleach cell i.e. image of cell before bleach. Middle panel, image of cell at the time of bleach. Right panel, image of post-bleach cell i.e. image of cell 30 sec after bleach. Photobleaching of GFP was performed with the 488-nm laser line at 30 mWatpower in a rectangular region of interest 32.85 µm2. The bleach areas are indicated by red rectangle. (TIF) [file pntd.0003367.s003.tif]

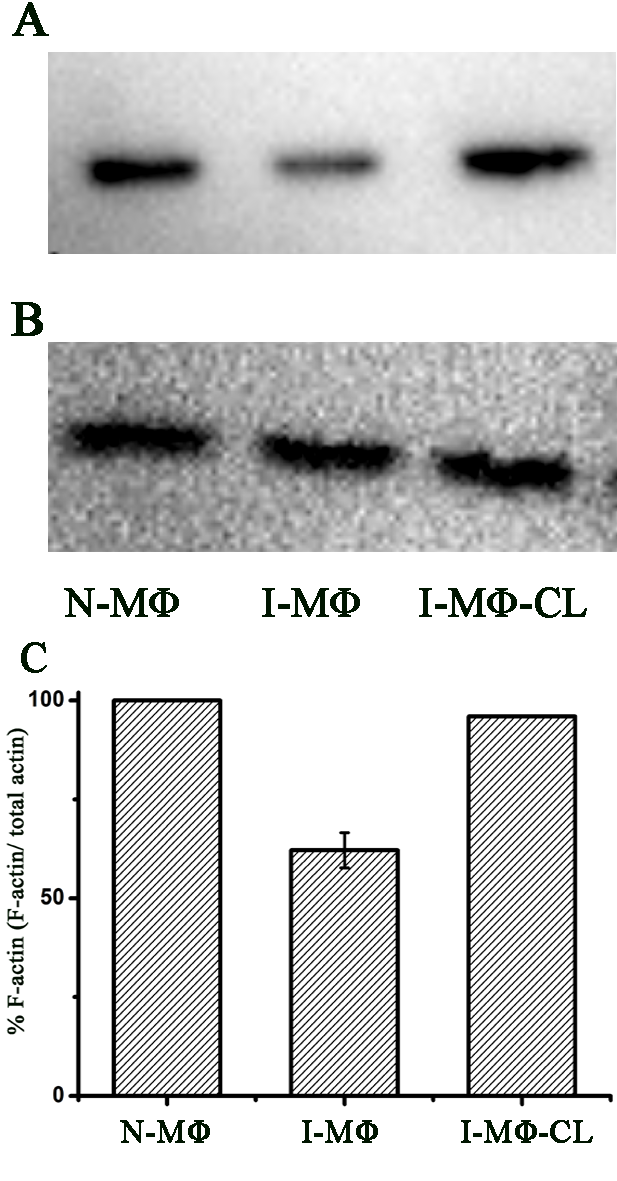

Supplement: Figure S5 — Immunoblot of actin. A) Expressions of F-actin. B) Expressions of total cellular actin. 106/ml cell were lysed in a RIPA buffer. 100 µg of cellular protein were used to detect F-actin and total cellular actin. C. The densitometry ratio of F-actin/total actin. The analysis was done by image-J. (TIF) [file pntd.0003367.s005.tif]
